# Telogen Effluvium: A Review of the Literature

**DOI:** 10.7759/cureus.8320

**Published:** 2020-05-27

**Authors:** Fahham Asghar, Nazia Shamim, Umar Farooque, Haris Sheikh, Ramsha Aqeel

**Affiliations:** 1 Neurology, Dow Medical College, Dow University of Health Sciences, Karachi, PAK; 2 Internal Medicine, Dow Medical College, Dow University of Health Sciences, Karachi, PAK; 3 Anaesthesiology, Aga Khan University, Karachi, PAK

**Keywords:** telogen effluvium, literature review, epidemiology, pathogenesis, diagnosis, treatment, alopecia, acute telogen effluvium, chronic telogen effluvium, causes of telogen effluvium

## Abstract

Telogen effluvium is one of the most common causes of alopecia. It is a scalp disorder characterized by excessive shedding of hair. Several factors such as drugs, trauma, and emotional and physiological stress can lead to the development of telogen effluvium. Multiple alterations in the hair cycle have been proposed as the underlying mechanism. Telogen effluvium can present as acute or chronic hair fall with symptoms such as trichodynia. Diagnostic tests that can be used include hair wash test, trichogram, phototrichogram, and scalp biopsy. In the treatment of telogen effluvium, it is essential to identify and remove the causative factors and to use drugs such as corticosteroids, minoxidil, and novel treatments such as CNPDA (caffeine, niacinamide, panthenol, dimethicone, and an acrylate polymer). Herein, we discuss the presentation, diagnostic approaches, and effective treatment options available for telogen effluvium.

## Introduction and background

Telogen effluvium is a scalp disorder characterized by diffuse, non-scarring shedding of hair [1]. The term “telogen effluvium” was proposed to differentiate it from excessive shedding of normal club hair. Various hypotheses are put forward regarding the pathophysiology of telogen effluvium. Headington proposed that there are five different functional types of telogen effluvium based on alternations in particular phases of the follicular cycle [2]. Whiting defined chronic telogen effluvium as an idiopathic disorder [3].

Telogen effluvium has been related to a variety of insults which can be physical, mental, or chemical in nature [1,4].

## Review

Epidemiology

Most of the cases of telogen effluvium are subclinical; therefore, its true incidence is not clearly known [5]. No racial predilection of the disease has been recognized and it affects both males and females, with a higher incidence rate in females. However, it should be taken into account that women take hair shedding problem more seriously than men and are likely over-represented in seeking medical treatment [1]. The association of telogen effluvium with age is unclear; however, elderly women are known to be more susceptible to acute telogen effluvium following fever, trauma, hemorrhage, or psychological stress [1]. Studies have reported the incidence of telogen effluvium in children to be around 2.7% [6].

Presentation

Acute Telogen Effluvium

Acute telogen effluvium is defined as hair shedding lasting for less than six months. Generally, hair loss occurs two to three months after the trigger exposure. In around 33% of the cases, the cause remains unknown [2]. Acute telogen effluvium usually undergoes remission in around 95% of cases. On examination of those with resolved effluvium, there is an appearance of shorter, re-growing frontal hair. Such hair can be seen in a large quantity using videodermoscopy [1,7]. A variant of acute telogen effluvium is telogen gravidarum, which is associated with pregnancy and usually occurs two to five months after childbirth [2].

Chronic Telogen Effluvium

Chronic telogen effluvium is a condition lasting for more than six months. The disorder mostly affects middle-aged women, having a prolonged fluctuating course. The examination of the scalp shows hair having normal thickness with signs of shorter re-growing hair in the frontal and bitemporal areas [1].

Pathogenesis

Telogen effluvium is caused by an abnormality in the normal hair cycle, which is triggered by numerous factors.

Normal Hair Cycle

A hair follicle has a three-phase life cycle. It consists of a growing phase (anagen), an involuting phase (catagen), and a resting phase (telogen). The anagen phase can last for about two to five years, and around 90% of scalp hair is in this phase [8]. The catagen phase is a much shorter phase, lasting three to six weeks. During this phase, the hair follicles go through a process of programmed cell death (apoptosis) [8,9]. Finally, the telogen phase lasts for around three to five months, and 10% of the scalp hair are in this phase. During this phase, the hair shaft matures into a club hair, which is eventually shed from the follicle. If the percentage of scalp follicles present in the telogen phase increases, this results in excessive shedding of hair [8,9].

Mechanism of Shedding

The five proposed mechanisms by which shedding of the hair may occur in telogen effluvium are as follows:

1. Immediate anagen release: This is due to an underlying cause. Follicles leave the anagen phase and enter the telogen phase prematurely, leading to increased shedding two to three months later [2].

2. Delayed anagen release: This is due to prolongation of the anagen phase resulting in heavy telogen shedding [2].

3. Short anagen syndrome: This is due to idiopathic shortening of the anagen phase, leading to persistent telogen effluvium. The pathogenesis behind most of the cases of chronic telogen effluvium is considered to be the short anagen syndrome [2].

4. Immediate telogen release: This is due to the shortening of the telogen phase, resulting in a massive release of club hair [2].

5. Delayed telogen release: This is due to a prolonged telogen phase and a delayed transition to anagen phase [2,10].

Causes of telogen effluvium

There are various factors that can initiate disturbance in the normal hair cycle.

Drugs

Numerous drugs can cause telogen hair loss and it usually starts after 12 weeks of dosage [2,4]. Changes in the dosage of drugs can also lead to excessive shedding [11]. Drugs that can cause telogen effluvium include oral contraceptive pills, androgens, retinoids, beta-blockers, ACE (angiotensin-converting enzyme) inhibitors, anticonvulsants, antidepressants, and anticoagulants (heparin) [11].

Physiological Stress

Increased physiological stress such as surgical trauma, high fever, chronic systemic illness, and hemorrhage can cause telogen effluvium [12]. Childbirth can also cause excessive hair to enter the telogen phase. This hair loss, telogen gravidarum, occurs approximately three months after childbirth [12].

Emotional Stress

The relationship between emotional stress and hair loss is ambiguous since hair loss itself is a source of emotional stress to the patient [11].

Medical Conditions

Numerous medical disorders can lead to telogen effluvium. Both hyper- and hypothyroidism can cause telogen effluvium, and this is reversed once the euthyroid state is achieved [13]. Chronic systemic disorders such as systemic amyloidosis, hepatic failure, chronic renal failure, inflammatory bowel disease, and lymphoproliferative disorders can also cause telogen effluvium [12]. It is also reported in some autoimmune diseases including dermatomyositis, chronic infections such as HIV, and secondary syphilis. Inflammatory disorders such as psoriasis and seborrheic dermatitis can also lead to diffuse telogen hair loss [11].

Dietary Triggers

Severe protein, fatty acid and zinc deficiency, chronic starvation, and caloric restriction can lead to telogen effluvium [11]. Essential fatty acid deficiency leads to telogen effluvium, and this usually occurs two to four months after insufficient intake [11,14]. Decreased body iron stores can cause it. However, this relationship is very controversial [14]. Vitamin D is vital for cell growth and, hence, its deficiency could also be a possible cause of it. Another cause can be biotin deficiency but is reportedly very rarely [11,15].

Ultraviolet Light

Researchers found an increased frequency of telogen effluvium between July and October. They hypothesized that it could be actinic effluvium, a summer effect, induced by sunlight and ultraviolet (UV) light, manifesting in autumn [16]. Electron microscopy of hair exposed to sunlight reveals alterations in the cellular components and damage to the hair cuticle and cortex. Both of these mechanisms can be attributed to increased shedding of hair in the telogen phase; however, it is not scientifically proven yet [1].

Diagnostic considerations

Trichodynia

A major symptom of telogen effluvium is trichodynia. It presents with complaints such as tenderness, pain, burning, itching, stinging, and diffuse alopecia [15,17].

Modified Wash Test and Hair Loss Count

The modified wash test is an office procedure that permits to identify patients with telogen effluvium or androgenetic alopecia, and the severity of diseases. It is performed after five days of abstention from shampooing. The patients are asked to wash and rinse their hair in a sink covered by gauze, collect the hair, let them dry, and put them in an envelope. Afterward, the collected hair are counted along with the percentage of vellus hair [18]. The results and possible diagnosis are as follows: 1. Telogen effluvium: More than 100 shed hair, less than 10% vellus.

2. Androgenetic alopecia: Less than 100 shed hair, more than 10% vellus.

3. Association of telogen effluvium and androgenetic alopecia: More than 100 shed hair, more than 10% vellus.

4. Normal or remitting telogen effluvium: Less than 100 shed hair, less than 10% vellus.

Trichogram

Trichogram is a plucking of hair in a defined area (40-60 hair). Cases of telogen effluvium show a significant reduction of the anagen:telogen ratio. More than 25% of hair are found to be in the telogen phase in the case of telogen effluvium [19].

*Phototrichogram and TrichoScan*®

This technique involves trimming the hair of a 2 sq. cm area of scalp, pictures of the same area taken on different days, and then compared in hair density, hair growth, and rate of shedding. Since only anagen hair would elongate it helps in the assessment of the ratio of anagen:telogen hair. A TrichoScan is a fully computerized phototrichogram [20]. A TrichoScan is a simpler, noninvasive, reproducible, and more sensitive than a classical trichogram and very useful in the diagnosis of hair loss [20].

Videodermoscopy

In the case of acute telogen effluvium, videodermoscopy will show numerous short re-growing hair with no variability of density [21].

Scalp Biopsy

It is recommended in cases where telogen loss lasts greater than six months. Performing multiple biopsies increases the diagnostic accuracy of telogen effluvium [22]. In the case of acute telogen effluvium, there is a normal to supernormal anagen:telogen ratio [23]. Follicular miniaturization and peribulbar infiltrate are not found. In chronic telogen effluvium, there are increased telogen hair, with an anagen:telogen ratio of 8:1 compared with 14:1 on normal scalp biopsies [21].

Management and treatment

Acute Shedding versus Chronic Shedding

Acute telogen effluvium becomes self-limited if the triggering factor is identified and removed. Causative conditions such as scalp conditions (e.g., psoriasis, seborrheic dermatitis) should be treated [24]. The patient’s drug history should be obtained in detail, and drugs suspected to cause the condition should be replaced or discontinued [5]. The longer the duration of shedding, the more probable the involvement of multiple and repetitive triggers such as nutritional deficiencies, thyroid disease, systemic illnesses, or infections. This makes the search for triggers more difficult and may require frequent visits [24].

Patient Education

Patient education is important in disease management. Disease correlation with triggers, and the timing of hair loss should be explained and frustrations addressed. Hair is an important part of the human body; the degree of psychological disability due to hair loss varies from person to person [25].

Correcting Deficiencies

If a measurable deficiency has been found, it should be corrected. A balanced diet and stable body weight are important. Although the use of polyphenolic compounds such as those in green tea has been reported to improve hair loss in mice, no such controlled studies are available for humans [26].

Minoxidil and Finasteride

The currently available FDA-approved standard drugs minoxidil and finasteride are neither efficient catagen inhibitors nor anagen inducers [5]. Catagen-inducing drugs (e.g., beta-blockers, retinoids, anticoagulants, antithyroid drugs) should be avoided, and catagen-inducing endocrine disorders (e.g., androgen disorders, thyroid disorders, abnormal prolactin levels) should be treated [1].

Topical Corticosteroids

Topical corticosteroids are employed by dermatologists in the treatment. If the patient reports decreasing trichodynia after the application of topical corticosteroids, it is a sign of the therapy being effective [27].

Systemic Corticosteroids

In chronic telogen effluvium, corticosteroids can be given systematically especially if telogen effluvium is the manifestation of underlying systemic disorder like SLE [28].

CNPDA

Davis et al. reported a novel treatment of thinning of hair. This new treatment named CNPDA comprises a combination of caffeine, niacinamide, panthenol, dimethicone, and an acrylate polymer. This combination leads to an increased cross-sectional area of individual terminal scalp hair by 10% [29].

## Conclusions

A thorough history, clinical examination, and laboratory investigations can aid in the diagnosis of telogen effluvium. Acute telogen effluvium can usually be resolved by removing the underlying causative factors. However, the treatment of chronic telogen effluvium can be challenging for physicians. A few treatment options are available, but standard guidelines for therapy, frequency, and dosage of drugs need to be established.
